# Bromodomain protein 4 discriminates tissue-specific super-enhancers containing disease-specific susceptibility loci in prostate and breast cancer

**DOI:** 10.1186/s12864-017-3620-y

**Published:** 2017-03-31

**Authors:** Verena Zuber, Francesco Bettella, Aree Witoelar, the PRACTICAL Consortium, Rosalind Eeles, Doug Easton, Zsofia Kote-Jarai, Ali Amin Al Olama, Sara Benlloch, Kenneth Muir, Graham G. Giles, Fredrik Wiklund, Henrik Gronberg, Christopher A. Haiman, Johanna Schleutker, Maren Weischer, Ruth C. Travis, David Neal, Paul Pharoah, Kay-Tee Khaw, Janet L. Stanford, William J. Blot, Stephen Thibodeau, Christiane Maier, Adam S. Kibel, Cezary Cybulski, Lisa Cannon-Albright, Hermann Brenner, Jong Park, Radka Kaneva, Jyotsna Batra, Manuel R. Teixeira, Hardev Pandha, the CRUK GWAS, the BCAC Consortium, Qin Wang, the TRICL Consortium, Ole A. Andreassen, Ian G. Mills, Alfonso Urbanucci

**Affiliations:** 1 Prostate Cancer Research Group, Centre for Molecular Medicine Norway (NCMM), Nordic EMBL Partnership, Faculty of Medicine, University of Oslo, Oslo, Norway; 2 NORMENT, KG Jebsen Centre for Psychosis Research, Institute of Clinical Medicine, University of Oslo, Oslo, Norway; 3 Division of Mental Health and Addiction, Oslo University Hospital, Oslo, Norway; 4 European Molecular Biology Laboratory, European Bioinformatics Institute, Wellcome Trust Genome Campus, Hinxton, Cambridge, UK; 5 Centre for Cancer Genetic Epidemiology, University of Cambridge, Cambridge, UK; 6 Department of Molecular Oncology, Institute for Cancer Research, Oslo University Hospital, Oslo, Norway; 7 PCUK Movember Centre of Excellence, CCRCB, Queen’s University, Belfast, UK

**Keywords:** BRD4, Genome-wide association studies, SNPs, Functional annotation, Chromatin, Risk loci, Prostate cancer risk, breast cancer risk, schizophrenia, super-enhancer

## Abstract

**Background:**

Epigenetic information can be used to identify clinically relevant genomic variants single nucleotide polymorphisms (SNPs) of functional importance in cancer development. Super-enhancers are cell-specific DNA elements, acting to determine tissue or cell identity and driving tumor progression. Although previous approaches have been tried to explain risk associated with SNPs in regulatory DNA elements, so far epigenetic readers such as bromodomain containing protein 4 (BRD4) and super-enhancers have not been used to annotate SNPs. In prostate cancer (PC), androgen receptor (AR) binding sites to chromatin have been used to inform functional annotations of SNPs.

**Results:**

Here we establish criteria for enhancer mapping which are applicable to other diseases and traits to achieve the optimal tissue-specific enrichment of PC risk SNPs. We used stratified Q-Q plots and Fisher test to assess the differential enrichment of SNPs mapping to specific categories of enhancers. We find that BRD4 is the key discriminant of tissue-specific enhancers, showing that it is more powerful than AR binding information to capture PC specific risk loci, and can be used with similar effect in breast cancer (BC) and applied to other diseases such as schizophrenia.

**Conclusions:**

This is the first study to evaluate the enrichment of epigenetic readers in genome-wide associations studies for SNPs within enhancers, and provides a powerful tool for enriching and prioritizing PC and BC genetic risk loci. Our study represents a proof of principle applicable to other diseases and traits that can be used to redefine molecular mechanisms of human phenotypic variation.

**Electronic supplementary material:**

The online version of this article (doi:10.1186/s12864-017-3620-y) contains supplementary material, which is available to authorized users.

## Background

Genome-wide association studies (GWASs) have linked more than ten thousand of single nucleotide polymorphisms (SNPs) to human diseases and traits [1]. Given that a great part of associated variants are located in known tissue-specific enhancers, a recent study by Tehranchi and colleagues [2] found that these non-coding variants affect transcription factors (TFs) binding and gene expression. Although they found that CCCTC-binding factor (CTCF) is likely to play a pioneering role in translating natural genetic variation in chromosomal architecture [2], we still strive to understand tumor-specific epigenetic features that render possible progression toward such disease. For instance, previous approaches have been adopted to explore disease risk association with regulatory DNA elements [3–6].

In prostate cancer (PC) the androgen receptor (AR) binds predominantly to gene-distal sites and has been used by multiple groups to functionally annotate genetic risk loci based on overlaps with risk single nucleotide polymorphisms (SNPs) as measured in genome-wide association studies (GWAS), which in some cases are also predicted to affect AR binding [7, 8].

Epigenetic marks such as acetylation on Histone 3 lysine 27 (H3K27ac) have been used as annotation of enhancers [9]. Moreover, regions of extended H3K27ac bound by combinations of mediator complex subunit 1 (MED1) and bromodomain containing protein 4 (BRD4) have been defined as super-enhancers important to determine cell identity [10–12]. BRD4 has proven to be involved in several diseases thanks to the small molecule inhibitor JQ1 [13]. In PC cells, BRD4 was recently shown to bind to the AR and affect its activity [14] while components of the mediator complex such as MED1 and MED12 were recently found to be implicated in advanced PC [15, 16].

SNPs associated with common diseases have been found to lie within enhancers driving transcriptional output and have been identified using different methods [9]. For PC, the most recent methods include genotyping matched to expression quantitative trait loci analysis and epigenetic marks such as H3K27Ac combined with chromatin accessibility [17, 18] or additional combination of binding information for key TFs such as AR and FOXA1 [19]. Here we combined information on H3K27ac profile with binding site data for BRD4 and MED12 to improve the functional annotation of PC risk SNPs based on a previously described enrichment analysis [18].

We show that this method is able to capture SNPs associated not only with PC but also in the context of Breast Cancer (BC) and Lung Cancer (LC) susceptibility. We find that BRD4 is the key discriminant of tissue-specific super-enhancers and binds disease specific PC and BC low *p-*value risk SNPs. Enrichment of disease-specific risk SNPs is higher when BRD4 binding profile information is incorporated with other epigenetic marks such as H3K27Ac and MED components, than for binding profiles of key TFs implicated in disease development and progression such as the AR or estrogen receptor (ER). Inhibitors for BRD4 are in clinical trials. However, little is known about the contribution of BRD4 to brain diseases. In order to evaluate if similar principles apply also for heritable mental disorders we extended our framework to epigenetic marks including BRD4 binding derived from Schwann cells and applied the enrichment analysis to GWAS studies of mental disorders from the Psychiatric Genetics Consortium (PGC) [20, 21].

## Methods

### Data source for enhancers’ annotation

AR binding information in both LNCaP and VCaP cells was retrieved from Massie et al., (2011) [22]. Raw data were aligned with novoalign to human genome version hg19, and peaks were called with MACS using default parameters after filtering low quality reads (score below 20). Resulting peaks were then overlapped using Bedtools. MED1 binding information and H3K27Ac profile in LNCaP cells was retrieved from Wang et al., (2012) [23] and re-analyzed as described above. To define the degree of overlap with super-enhancers, we also downloaded super-enhancers coordinates from dbSUPER database [24]. BRD4 binding information and H3K27Ac profile in VCaP cells was retrieved from Asangani et al., (2014) [14]. ER and BRD4 binding information were retrieved from Nagarajan et al., (2014) [25]. H3K27Ac profile in MCF7 was retrieved from Theodorou et al., (2013) [26]. BRD4 and MED1 binding information, and H3K27Ac profile for small cell lung cancer (SCLC) cell line H2171 and Schwann cells were retrieved from cistrome [27]. All cell-specific datasets were equally analyzed to ensure comparability within a tissue type.

Enhancers were defined in LNCaP based on (1) extended H3K27Ac marked regions ranging from 3000 bp to 200 kb (Additional file 1); (2) an intersection of these H3K27Ac marked regions with MED12 binding sites (Additional file 2). In VCaP cells enhancers were defined (3) as an extended H3K27Ac marked regions ranging from 3000 bp to 200 kb (Additional file 3) (4) the intersection of H3K27Ac stretches longer than 2000 bp and BRD4 binding sites (in VCaP cultured in presence of androgens) (Additional file 4) or (5) as BRD4 sites alone (Additional file 5). (6) To achieve a consensus map of super-enhancers in PC (Additional file 6) we selected super-enhancers found in LNCaP cells that were found to have H3K27Ac and BRD4 binding also in VCaP cells.

Enhancers in MCF7 cells were identified following the criteria described in Hnisz et al., (2013) [12]. First, H3K27Ac peaks closer than 100 bp were merged, then only stretches longer than 2000 bp were selected (Additional file 7). Different compendia of enhancers were then created based on the presence of BRD4 (Additional file 8) and ER binding (Additional file 9) or the combination of these features (Additional files 10 and 11). The same type of algorithm was followed to identify enhancers in H2171 and Schwann cells 90-8TL (Additional files 12, 13, 14, 15, 16, 17 and 18). DNase I hypersensitive sites (DHS) profiles for LNCaP cells were retrieved from He et al. (2012) [28] and from ENCODE (Additional files 19 and 20). A more stringent profile of these two based on overlap (Additional file 21) was also included.

### Data source for summary statistics of genome-wide association studies

We obtained summary statistics from large meta-analyses of the traits of interest. In particular, the summary statistics for association with PC risk were obtained from the Illumina array Collaborative Oncological Gene-environment Study (iCOGS) consortium [29] and comprised information on 25,074 cases and 24,272 controls genotyped on a customized array including 211,155 SNPs. Additionally, we used summary statistics on 525,821 SNPs for association with PC risk derived from a smaller UK-based cohort including 1854 cases and 1854 controls in collaboration with the PRACTICAL consortium [30]. Genetic association with BC risk was obtained in collaboration with the BCAC consortium and was derived from a meta-analysis including 15,863 cases and 40,022 controls on ~2.5 million SNPs [31]. We collected also summary statistics for 14,900 cases of lung cancer (LC) and 29,485 controls including 2,433,836 SNPs from the TRICL consortium [32]. From the IGAP consortium we obtained summary data from 17,008 Alzheimer's disease cases and 37,154 controls genotyped on 518,871 SNPs [33]. Finally from the PGC consortium we used summary statistics on association with schizophrenia on 36,989 cases and 113,075 controls including 2,540,803 SNPs [21], and summary statistics on association with bipolar disorder on 11,974 cases and 51,792 controls on a total of 2,382,073 SNPs [20].

### SNPs enrichment method

Enrichment is defined by the presence of lower *p-*values than expected by chance. Quantile-quantile (Q-Q) plots are tools commonly used in genetics to visualize enrichment [18]. Typically, the observed *p*-value quantiles on the *y-*axes are plotted against the theoretical *p*-value quantiles under the assumption of no association (i.e. following the quantiles of the uniform distribution) on the *x-*axes. In case of no association, a Q-Q plot follows a straight 0–1 line starting from the origin. In the presence of association, the enrichment (of low *p*-values) is described by the deflection of the Q-Q plot from this theoretical line of no association. We used stratified Q-Q plots to assess differential enrichment of SNPs mapping to specific categories of enhancers. Stratified Q-Q plots have been used previously to demonstrate enrichment of general location annotation categories such as 5’UTR SNPs [18].

### Quantifying SNPs enrichment within sets of enhancers

To assess the significance of the association enrichment among the sets of SNPs within enhancers we used Fisher’s hypergeometric test. More specifically, we tested for over-representation of genome-wide significant SNPs (i.e. association of –log10 *p*-value > 7.3) within specific enhancers. We adjusted for multiple testing using a Bonferroni-correction accounting for the number of annotations tested.

### Random pruning

The statistical models underlying the SNP enrichment analyses carried out here generally assume independence of the data. Far from resembling independent samples, SNPs are linked by complex correlation patterns reflected in their linkage disequilibrium (LD) structure. In order to adhere more closely to the independence assumption, and to rule out bias due to confounding factors such as LD, and assess whether the intrinsic capacity of functional annotations to enrich specific SNP sets was due to such confounding factors, the SNPs were randomly pruned prior to the analyses by randomly selecting representatives from all 1Mbase LD blocks of SNPs with pairwise *r* ^2^ ≥ 0.2. Iterating the random pruning procedure 100 times and subsequently averaging the corresponding test statistics compensated the arbitrariness in the choice of representative SNPs. These analyses were performed and shown in Additional file 22: Figures S1, S3, and S6.

## Results

To assess whether tissue or cell-specific enhancers could mark tissue-specific risk SNPs associated with development of PC, we analyzed datasets from two studies that profiled MED12 binding and H3K27Ac map in LNCaP cells [23], and BRD4 and H3K27Ac in VCaP cells [14]. MED12, is a subunit of the same chromatin looping mediator complex as MED1 [34] therefore we used it for our PC study assuming that these two subunits would have similar binding profiles in the same cells.

**Fig. 1:**
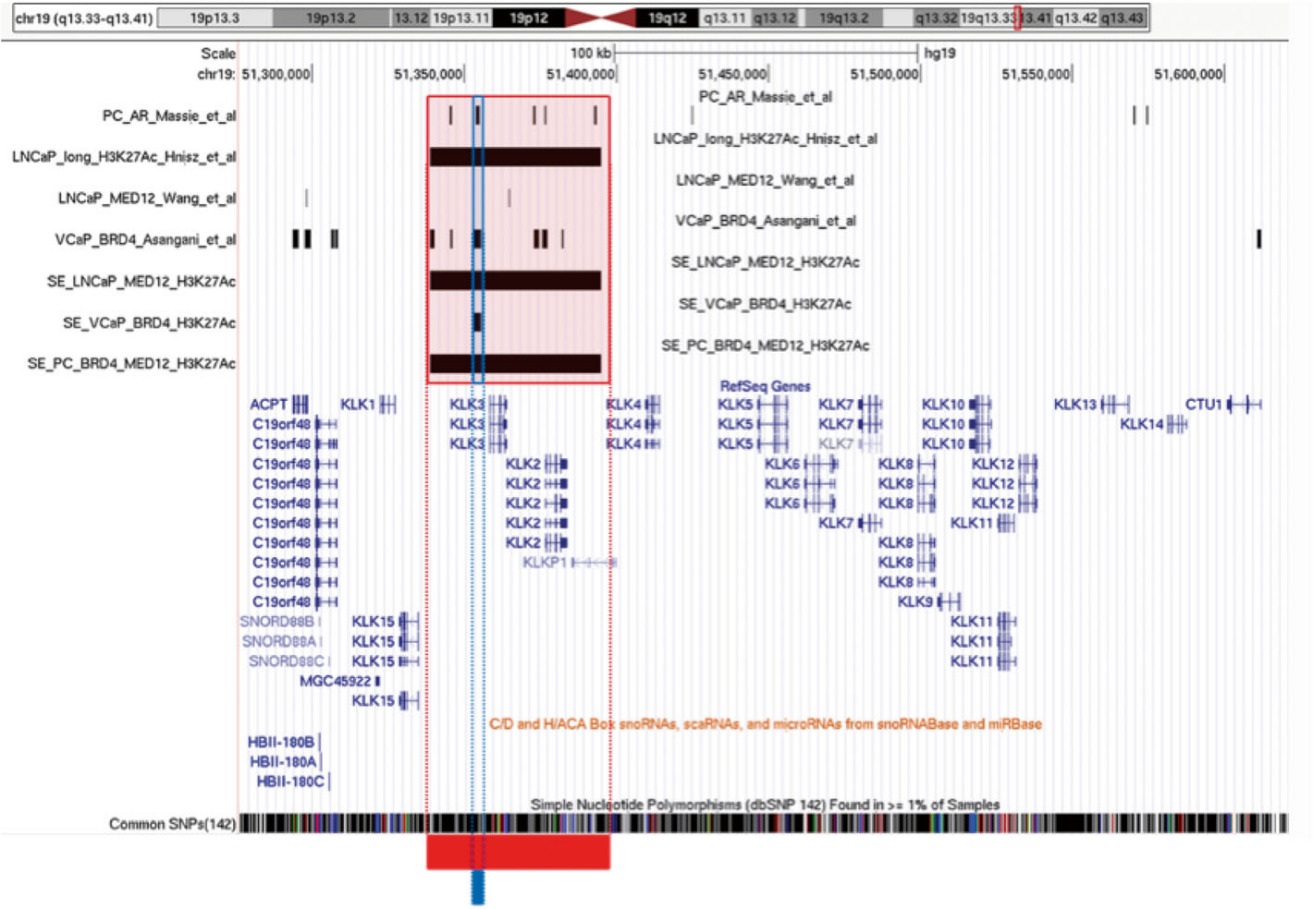
Definition of enhancers using chromatin marks and generic epigenetic readers. UCSC genome browser snapshot of the kallikreins locus showing enhancers identified in LNCaP based on MED12 binding information retrieved from Wang et al., (2012), and H3K27Ac profile retrieved from Hnisz et al., (2014) [12]; Enhancers identified in VCaP based on BRD4 binding and H3K27Ac retrieved from Asangani et al., (2014) [14]; and common enhancers in prostate cancer (PC) identified selecting enhancers in LNCaP which also had BRD4 and acetylation signature according to the compendium of enhancers in VCaP cells. In the locus shown here the long stretch of H3K27Ac includes also MED12 according to Wang et al., (2011) [23] and BRD4 binding sites. At the bottom of the figure SNPs within these particular enhancers are indicated with the red line for SNPs found in the enhancers in LNCaP cells and PC common enhancers, and with the blue line for SNPs found in the enhancer in VCaP cells. Independent tracks for the androgen receptor (AR) binding sites in common in LNCaP and VCaP cells according to Massie et al., (2011) [22] re-analyzed for this study are also shown

Enhancers were defined in LNCaP based on (1) extended H3K27Ac marked regions (Additional file 1); (2) an intersection of these H3K27Ac marked regions with MED12 binding sites (Additional file 2). In VCaP cells enhancers were defined as (3) extended H3K27Ac marked regions (Additional file 3) (4) the intersection of H3K27Ac stretches and BRD4 binding sites (Additional file 4) or (5) as BRD4 sites alone (Additional file 5). (6) To achieve a consensus map of enhancers in PC (Additional file 6) we intersected the enhancers found in both LNCaP and VCaP cells characterized by all three epigenetic features and responded to the definition of super-enhancers [12] (Table 1 and Fig. 1).

**Table 1:** SNPs mapping to epigenetic marks used to define enhancers in prostate and breast cancer

Cell line/Tumor type	Epigenetic marks	Key transcription factor^a^	Number of SNPs within the intervals covered by the array:			
			iCOGS	PRACTICAL	BCAC	All
LNCaP	H3K27Ac	----	1605	3092	13482	13503
		AR	669	1274	5664	5671
LNCaP	H3K27Ac + MED12	----	685	1271	5428	5442
		AR	279	541	2310	2316
VCaP	H3K27Ac + BRD4	----	587	983	4218	4239
		AR	342	502	2148	2154
VCaP	BRD4	----	859	1595	7984	8066
		AR	23	60	233	233
VCaP	H3K27Ac	----	3896	8440	38150	38444
PC	H3K27Ac + MED12 + BRD4	----	82	130	618	619
		AR	49	46	248	249
PC	----	AR	496	1403	5950	5967
MCF7	H3K27Ac + BRD4	----	8783	21058	93969	94858
		ER	4296	10607	45937	46222
MCF7	H3K27Ac	----	19270	48028	215997	217382
		ER	6710	16206	69743	70058
MCF7	BRD4	----	280	495	2617	2641
		ER	34	66	282	282
MCF7	----	ER	158	371	1638	1639

^a^Binding information for key transcription factors such as androgen receptor (AR) or estrogen receptor (ER) where used alone or in combination with the epigenetic mark profiles in order to assess their capacity to refine enrichment of risk SNPs

### Enrichment of SNPs associated with prostate cancer in regions bound by MED and BRD4, marked by H3K27Ac in prostate cancer cells

**Fig. 2:**
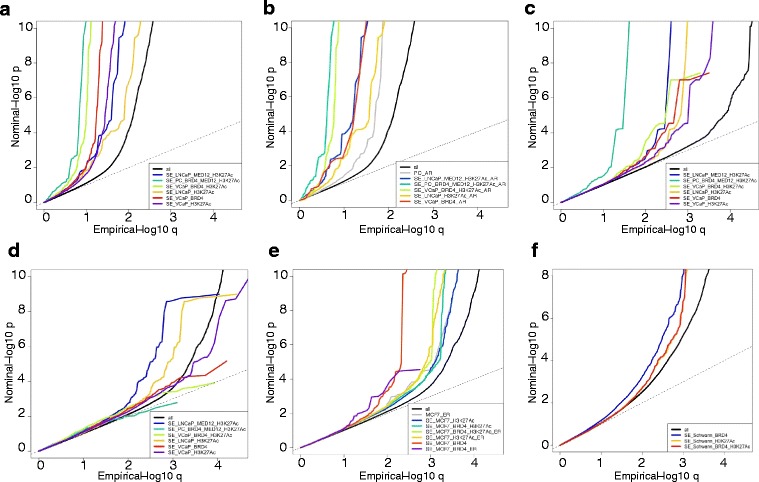
Enrichment of SNPs lying within enhancers. Q-Q plots visualizing the *p*-value enrichment of sets of SNPs mapping within genomic intervals identified as regions of putative enhancers or key transcription factor binding sites. The *p*-values describe the association of a specific SNP with prostate (iCOGs in panel **a** & **b**; PRACTICAL in panel **c**) and breast cancer (BCAC in panel **d** & **e**). The genomic intervals represent regions bound by MED12, BRD4 with a H3K27Ac modification in prostate cancer cell lines (LNCaP and VCaP), or in overlapping regions profiled for a combination of the features in the prostate cancer (PC) cell-lines as indicated (**a**, **c**, **d**), intersected with AR binding sites (**b**), or regions found in MCF7 (**e**), as indicated in the legends. (**f**) Q-Q plots visualizing the *p*-value enrichment of schizophrenia associated SNPs (PGC) lying within enhancers identified in Schwann cells

First, we overlaid genome coordinates of enhancers in PC cell lines, as defined previously, with genomic coordinates of all SNPs in the PC iCOGS dataset [29]. To visualize differential enrichment patterns of specific epigenetic markers with respect to their genetic association with PC risk we generated stratified Q-Q plots which is a method for visualizing the enrichment of statistical association relative to that expected under the global null hypothesis [18]. Q-Q plots show that SNPs within regions with different genomic features (H3K27ac, BRD4, and MED12, or a combination of these) had different enrichment patterns compared to all SNPs (Fig. 2a). The SNPs contained in common PC enhancers, and therefore characterized by BRD4 and MED12 binding, and a long stretch of H3K27Ac had lower *p-*values than SNPs contained in enhancers identified in VCaP cells by mapping long stretches of H3K27Ac and BRD4 binding. SNPs associated with PC risk were more enriched within BRD4 binding sites alone than within H3K27Ac sites or H3K27Ac/MED12 overlapping sites in LNCaP. In addition, we focused on SNPs achieving genome wide significance and compared overrepresentation of these SNPs mapping to the above-described enhancers (Additional file 22: Table S1). 12% and 3% of the SNPs contained in PC enhancers achieved genome-wide significance in the iCOGS and in the PRACTICAL GWAS respectively. SNPs that achieved significance in iCOGs are listed in Additional file 22: Table S2. These results highlight that combining generic epigenetic marks such as H3K27Ac with generic epigenetic readers such as BRD4 and with MED binding increases the capacity of capturing SNPs associated with PC.

Importantly, to rule out possible confounding factors, we first randomly pruned the SNPs, selecting one representative SNP per LD block. The random pruning did not change the enrichments patterns caused by the functional annotations (Additional file 22: Figure S1). Secondly, in order to rule out that the enrichment merely results from the non-independence of the SNPs in the enhancer regions or other confounding features of these, we compared the observed enrichment to the one attained on a set of SNPs numerically matching those in the enhancer regions on minor allele frequencies and mutual LD *r* ^2^ (Additional file 22: Figure S2). The numerically matched SNP set was also used as control set to assess the enrichment significance (Additional file 22: Table S1, S3, S4) by means of Fisher’s hypergeometric test (see Methods).

### Enrichment of prostate cancer associated SNPs within androgen receptor binding information.

We also compared the genomic coordinates of the SNPs to the coordinates for AR binding sites (ARBSs). Despite the use in the literature of ARBSs for functional annotation of GWAS significant PC SNPs, intersecting enhancer information with AR binding data did not lead to any further enrichment of SNPs associated with PC compared with enhancer information alone (Fig. 2b and Additional file 22: Table S3). In particular, although intersecting AR binding information induced a slight left-shift of the Q-Q plot for enhancers marked by H3K27Ac, MED12, and BRD4 binding, and for enhancers marked by H3K27Ac and BRD4, the enrichment was caused by the same SNPs responsible for the enrichment without AR binding information (see Additional file 22: Table S1 and S3). Furthermore, enhancer information outperformed ARBSs profile alone, or in combination with H3K27Ac profile, in enriching for genome-wide significant *p-*valued SNPs in PC (Additional file 22: Table S3), and overlapping AR with BRD4 binding sites did not alter the superior capability of BRD4 (as in Fig. 2a) to enrich for disease associated SNPs. Interestingly, although DHSs have been used to predict locations of common disease-associated variation [3], DHSs profiles enriched less than ARBSs alone (Additional file 22: Figure S3).

### Validation of the enrichment method on an independent GWAS for prostate cancer.

Finally, we validated our results on the independent PC GWAS obtained from the PRACTICAL consortium measured on a smaller UK-based cohort [30] (Fig. 2c). Again, we observed the strongest SNP enrichment in PC super-enhancers marked by H3K27Ac, MED12, and BRD4 binding.

### BRD4 binding sites derived from prostate cancer cells do not enrich for SNPs associated with breast cancer

To test the specificity of BRD4, MED12 and H3K27Ac profiles in PC cells in identifying tissue-specific SNPs, we performed a similar enrichment analysis for genetic association with BC risk measured on the genotype array content from the BCAC [31] (Fig. 2d). Enhancers defined on the basis of BRD4 binding profile in PC cells failed to enrich specifically for BC associated SNPs. Whilst H3K27ac and MED12 together achieved some enrichment of BC SNPs, the addition of BRD4 depleted this enrichment entirely. Importantly, once again, randomly pruning the SNPs did not alter the results of the enrichment analysis (Additional file 22: Figure S4). These results are in stark contrast to the analysis on PC datasets in which inclusion of BRD4 enhanced enrichment of low *p-*valued SNPs associated with PC, and suggests a hierarchical determination of tissue-specificity, based on the subsequential deposition of these epigenetic marks. Taken together, this indicates that BRD4 substantially contributes to prostate-specific SNP enrichment within super-enhancers.

Of note, the genomic distribution of the BCAC SNP array mirrored the genomic distribution of the SNP arrays used for iCOGS with the majority of SNPs located within intronic (48% and 57%, respectively) and intergenic (48% and 34%, respectively) regions of the genome (Additional file 22: Figure S5) thus meaning that whilst the number of SNPs differed between the PC and BC studies, there was no genomic distribution bias for imputed SNPs. The SNPs included within the enhancers defined in this study reflected similar distributions, with the only exception of SNPs lists derived from LNCaP cells that were slightly biased toward intergenic regions. Around 69% to 77% of the SNPs were located within intergenic regions (data not shown).

### Enrichment of SNPs associated with breast cancer in regions bound by BRD4, marked by H3K27Ac in breast cancer cells

Next, we sought to identify whether using BC-specific epigenetic profiles for the same markers derived from the BC cell line MCF7, we would be able to repeat the same performance as in the PC enrichment analysis. Therefore we retrieved genome-wide profiles of H3K27Ac, ER, and BRD4 binding in MCF7 [25], compiled a similar list of enhancers (Table 1 and Additional file 22: Figure S6), and performed an enrichment analysis of association with BC risk on the BCAC GWAS (Fig. 2e and Additional file 22: Table S4). Information on MED binding is not available for BC cell lines. However, BRD4 binding information alone caused the strongest enrichment of SNPs associated with BC (Additional file 22: Table S5). These data confirm that BRD4 alone is an important enhancer and super-enhancer discriminant, which binds disease-specific susceptibility loci in a tissue specific fashion. Randomly pruning the SNPs involved, proved not to alter the capacity of BRD4 of capturing disease-specific associated SNPs (Additional file 22: Figure S7). Interestingly, pruning the SNPs revealed that ER capability to capture disease associated SNPs in combination with other epigenetic features was enhanced, possibly suggesting a different contribution of ER and AR in breast and PC pathogenesis, respectively.

As counterproof, we tested whether BC epigenetic profiles caused any enrichment in iCOGS PC associations, but no such enrichment was detected (Additional file 22: Figure S8). These results are consistent with BRD4 binding being cell and tissue-specific [35]. Moreover, these results pinpoint the tissue-specificity of risk loci and hint that BRD4 activity may be influenced by genetic variations as it is for TFs [2].

### Enrichment of risk SNPs associated with lung cancer and psychiatric traits using H3K27Ac profiles, BRD4, and MED binding sites derived from relevant cell lines

To understand whether the properties of BRD4 binding to clinically relevant genetic risk loci is confined to PC and BC only, or such selectivity can also be observed to other diseases and traits, we retrieved binding information for BRD4, MED1 and H3K27Ac profiles available for the lynphoblastoid cell line H2171 derived from a metastatic site in a LC patient [10] and from the malignant peripheral nerve sheath tumor Schwann cells 90-8TL [36] (Additional files 12, 13, 14, 15, 16, 17 and 18). To retrieve associations of these epigenetic features with other phenotypes we collected summary statistics for LC [32], Alzheimer’s disease [33], schizophrenia [21], and bipolar disorder [20].

BRD4 binding information alone caused the strongest enrichment of associations with LC, although combined information for BRD4 and MED1 binding, also combined with H3K27Ac profile failed to improve the enrichment of low *p*-value SNPs (Additional file 22: Figure S9a). We speculate that the LC cell line H2171 might not reflect characteristics of the tissue of origin, as well as the PC and BC cell lines. However, upon assessing the enrichment using epigenetic features related to PC cells for the same LC GWAS (Additional file 22: Figure S9b), as expected, we detected none, confirming that BRD4 binding information in H2171 retains some tissue-specificity and capacity to enrich for LC tissue-specific risk SNPs.

Next, we applied our enrichment method to perform an inverse analysis in which we sought to understand whether any association could be found between epigenetic features related to Schwann cells (the only brain cells for which H3K27Ac profile and BRD4 binding information were publicly available) and three diseases affecting the brain. No enrichment for SNPs associated with Alzheimer disease and bipolar disorder was detected (Additional file 22: Figure S10a&b). However, low *p*-valued SNPs associated with schizophrenia were highly enriched within BRD4 binding sites (Fig. 2f). Interestingly H3K27Ac profiles also enriched substantially for clinically relevant SNPs associated with schizophrenia. These data suggest that BRD4 activity in Schwann cells could potentially be involved in the etiology of schizophrenia [37], and grant further investigation on the molecular mechanism underlying these findings.

## Discussion

**Fig. 3:**
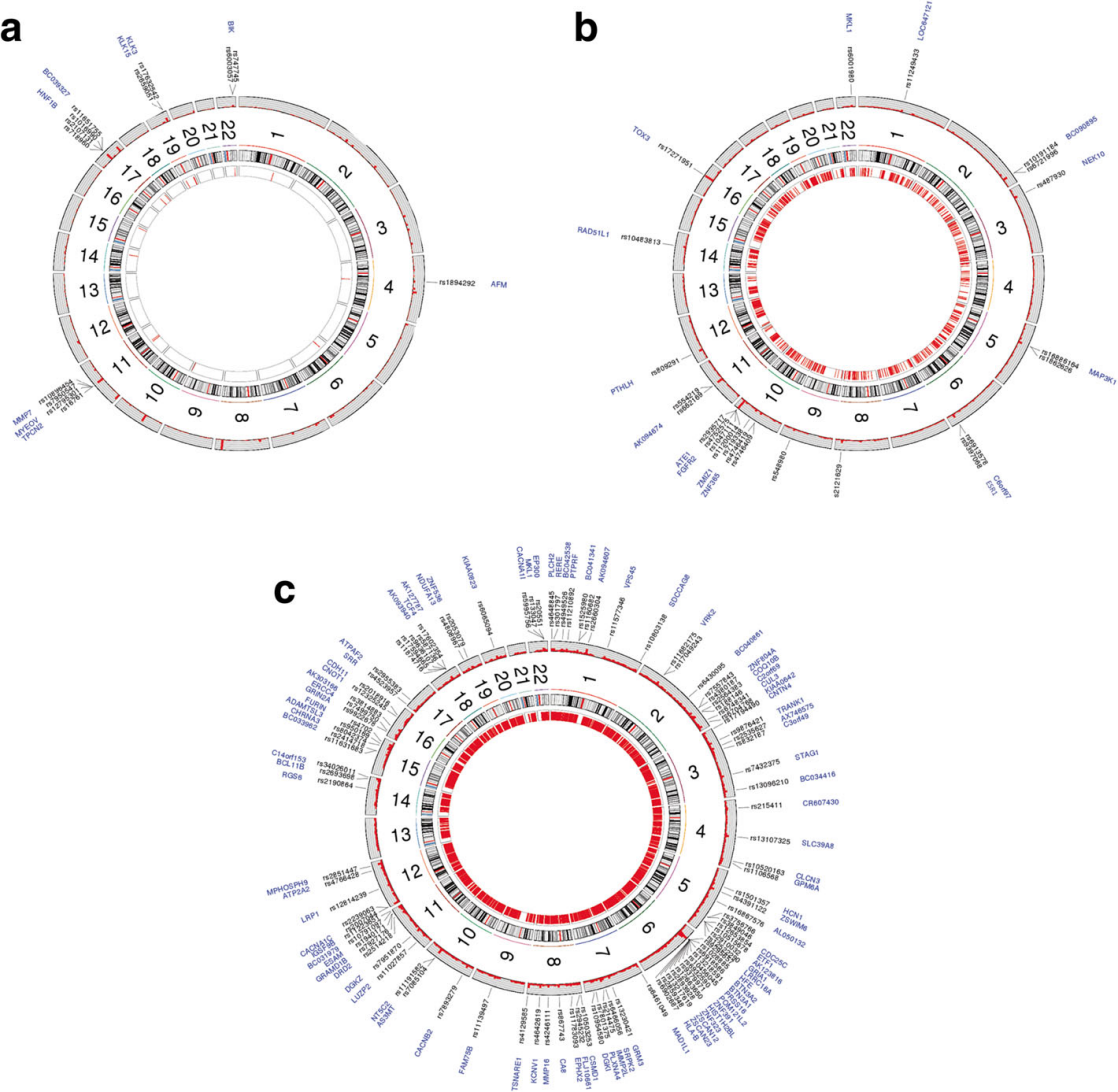
Circular plots of GWAS significant SNPs overlapping with putative super-enhancers. The outmost circles depict chromosome-wise histograms showing p-values of SNP loci (LD r^2 < 0.2 within 1Mbase) representatives for SNPs in iCOGS (**a**), for SNPs in BCAC (**b**), and for SNPs associated with schizophrenia according to PGC (**c**). GWAS-significant SNPs are labeled and the nearby genes are also indicated. The inmost circle represents super-enhancers regions identified in prostate cancer cells (SE_PC_BRD4_MED12_H3K27Ac) (**a**), breast cancer cells (SE_MCF7_BRD4) (**b**), and in Schwann cells (SE_Schwann_BRD4) (**c**) that were most enriched of low p-value SNPs

With the discovery of significant numbers of cancer genetic risk loci through GWAS there is now a major focus on the functional annotation of these loci to prioritize them for further biological study. So far this annotation has been undertaken Post-GWAS and has often employed classifiers of open chromatin, for example DHS, as a primary annotation followed by genome-wide binding maps for tissue-specific transcription factors such as the AR for PC or the ER for BC, while combining this information with H3K27Ac and open chromatin in a tissue-specific manner [38]. In this study we ask whether it is possible to use binding sites data and chromatin marks upfront to enrich for genetic risk factors in a cancer type-specific manner. We show that an enhancer signature comprising a number of factors but dominated by BRD4 allows for the enrichment of PC-specific and BC-specific genetic risk loci (Fig. 3a and b ). Interestingly, these chromatin features have been previously reported to be characteristic of super-enhancer-like profiles [10–12, 35]. We found a strong degree of tissue-specificity, that is when profiles are derived from cell-lines associated with specific cancer types such as the cancer of the breast and prostate, they become far more effective at enriching for cancer-type specific risk loci than other widely used cancer type-specific TFs such as the AR, ER or DHSs alone. We also applied this enrichment strategy to infer that BRD4 binding information may allow in future for the upfront nomination of genomic-regions for high-coverage sequencing in risk studies for schizophrenia (Fig. 3c). Functional determination of the impact of risk SNPs have been the priority of several consortia aiming to uncover the effects on epigenetics mediated by clinically relevant risk variants located in non-exonic regions [39]. Our study implies a conserved and important relationship between enhancers and cancer-associated risk loci, which is being pinpointed also by recent work linking the effect of genetic variation to TFs binding [2]. Our approach is the first one that implies an effect of such genetic variations on the activity of generic epigenetic readers. This is also the first time that such epigenetic readers have been evaluated as enrichment factors for SNPs without prior filtering based on published *p-*values for risk association.

We highlight the possibility that SNPs lying within super-enhancers marked by BRD4 are more likely to be associated with an increased susceptibility to BC, PC, and schizophrenia. The expression of the genes regulated by enhancers identified in these diseases could be altered by the presence of specific SNPs lying therein (Additional file 22: Figure S11). This is a concept that has recently been postulated for cancer mutations occurring in a chromatin-specific context [40].

## Conclusions

**Fig. 4:**
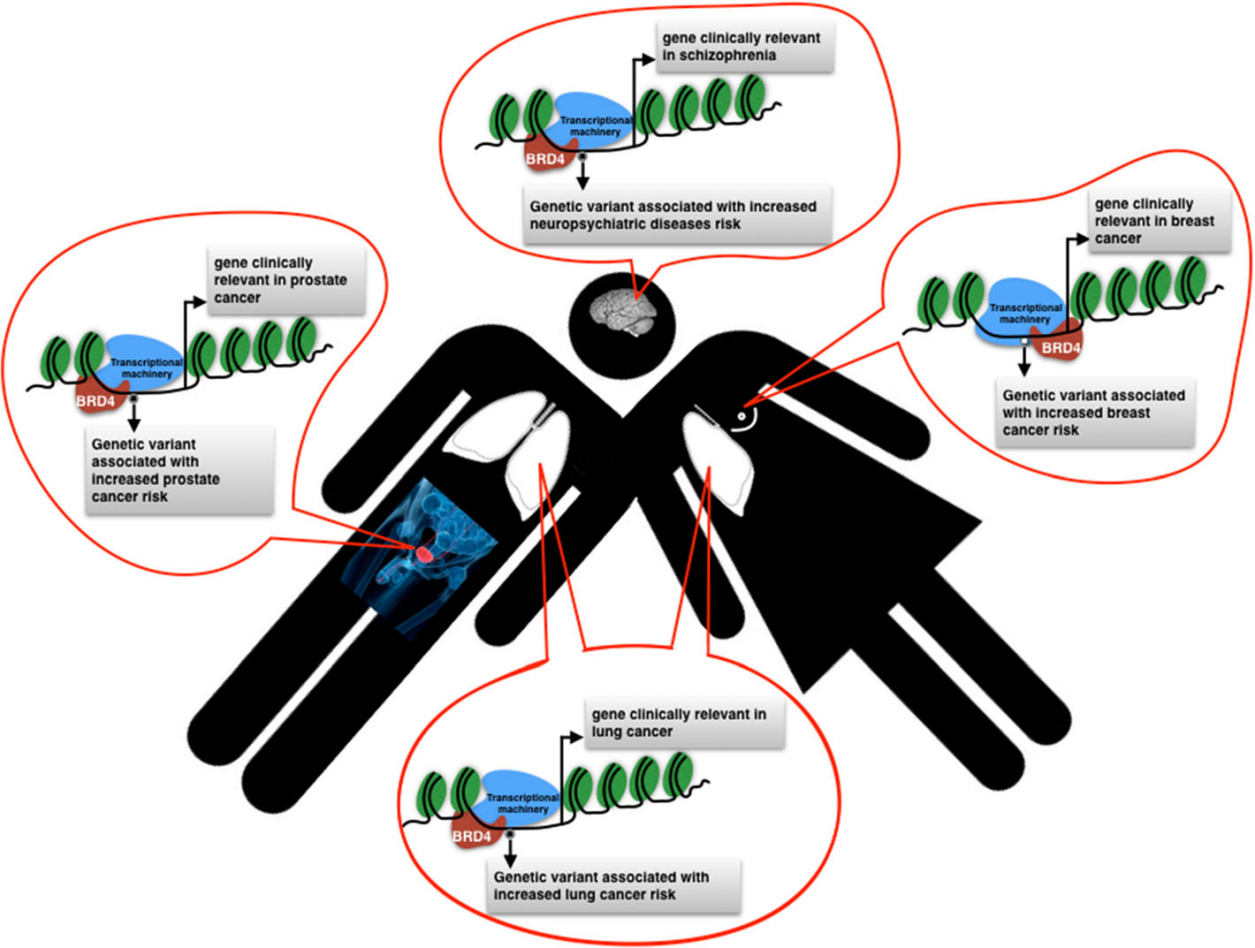
Tissue-specific super-enhancers usage and identification of clinically relevant genetic variations associated with diseases and traits. The method for prioritization of clinically relevant SNPs is based on the identification of risk SNPs with GWAS significance that are associated with BRD4 binding to chromatin, within tissue-specific super-enhancers rather transcription factors binding

In conclusion we have discovered that BRD4-bound super-enhancers provide a powerful tool for enriching and prioritizing PC and BC genetic risk loci (Fig. 4), and have shown that key TFs such as AR or ER, despite being pivotal tissue-specific TFs, do not contribute to tissue-specific genetic risk enrichment more than epigenetic factors. We propose to refine disease specific risk loci enrichment with the identification of potential binding of BRD4 combined with key MED components and acetylation profiles. Our study will promote the use of BRD4 for SNP annotation as the genetic landscape for different diseases goes on expanding.

## Additional files

Additional file 1: SE_LNCaP_H3K27Ac. (XLS 84 kb)

Additional file 2: SE_LNCaP_MED12_H3K27Ac. (XLS 45 kb)

Additional file 3: SE_VCaP_H3K27Ac. (BED 344 kb)

Additional file 4 SE_VCaP_BRD4_H3K27Ac. (XLS 42 kb)

Additional file 5: SE_VCaP_BRD4. (XLS 945 kb)

Additional file 6: SE_PC_BRD4_MED12_H3K27Ac. (XLS 28 kb)

Additional file 7: SE_MCF7_H3K27Ac. (XLS 539 kb)

Additional file 8: SE_MCF7_BRD4_ER. (XLS 72 kb)

Additional file 9: SE_MCF7_H3K27Ac_ER. (XLS 87 kb)

Additional file 10: SE_MCF7_BRD4_H3K27Ac_ER. (XLS 54 kb)

Additional file 11: SE_MCF7_BRD4_H3K27Ac. (XLS 137 kb)

Additional file 12: SE_H2171_BRD4_MED1_H3K27Ac. (BED 53 kb)

Additional file 13: SE_H2171_BRD4. (BED 1857 kb)

Additional file 14: SE_H2171_H3K27Ac. (BED 88 kb)

Additional file 15: SE_H2171_MED1_H3K27Ac. (BED 53 kb)

Additional file 16: SE_Schwann_BRD4_H3K27Ac. (BED 153 kb)

Additional file 17: SE_Schwann_BRD4. (BED 916 kb)

Additional file 18: SE_Schwann_H3K27Ac. (BED 195 kb)

Additional file 19: DHS_consensus. (BED 3694 kb)

Additional file 20: DHS_encode. (BED 6397 kb)

Additional file 21: DHS_He. (BED 4673 kb)

Additional file 22: Including Supplementary Material such as Supplementary **Figures S1–S11**, Supplementary **Tables S1–S5**, and Supplementary References. (DOCX 2037 kb)

## Abbreviations

AR: Androgen receptor
ARBSs: AR binding sites
BC: Breast cancer
BCAC: Breast Cancer Association Consortium
BRD4: bromodomain containing protein 4
ENCODE: Encyclopedia of DNA Elements
ER: Estrogen receptor
GWAS: Genome-wide association studies
H3K27Ac: Acetylation on Histone 3 lysine 27
iCOGS: Illumina array Collaborative Oncological Gene-environment Study
MED1/MED12: Mediator complex subunit 1/12
PC: Prostate cancer
PRACTICAL: Prostate Cancer Association Group to Investigate Cancer Associated Alterations in the Genome
SNPs: Single nucleotide polymorphisms

## References

[1] Welter D, MacArthur J, Morales J, Burdett T, Hall P, Junkins H, Klemm A, Flicek P, Manolio T, Hindorff L (2014). The NHGRI GWAS Catalog, a curated resource of SNP-trait associations. Nucleic acids research.

[2] Tehranchi AK, Myrthil M, Martin T, Hie BL, Golan D, Fraser HB (2016). Pooled ChIP-Seq Links Variation in Transcription Factor Binding to Complex Disease Risk. Cell.

[3] Maurano MT, Humbert R, Rynes E, Thurman RE, Haugen E, Wang H, Reynolds AP, Sandstrom R, Qu H, Brody J (2012). Systematic localization of common disease-associated variation in regulatory DNA. Science (New York, NY).

[4] Coetzee SG, Shen HC, Hazelett DJ, Lawrenson K, Kuchenbaecker K, Tyrer J, Rhie SK, Levanon K, Karst A, Drapkin R et al.: Cell Type Specific Enrichment Of Risk Associated Regulatory Elements At Ovarian Cancer Susceptibility Loci. Human molecular genetics. 2015;24(13):3595–607. 10.1093/hmg/ddv101 PMC4459387 25804953

[5] Paul DS, Soranzo N, Beck S (2014). Functional interpretation of non-coding sequence variation: concepts and challenges. Bioessays.

[6] Ritchie GR, Dunham I, Zeggini E, Flicek P (2014). Functional annotation of noncoding sequence variants. Nat Methods.

[7] Huang CN, Huang SP, Pao JB, Chang TY, Lan YH, Lu TL, Lee HZ, Juang SH, Wu PP, Pu YS (2012). Genetic polymorphisms in androgen receptor-binding sites predict survival in prostate cancer patients receiving androgen-deprivation therapy. Ann Oncol.

[8] Hazelett DJ, Rhie SK, Gaddis M, Yan C, Lakeland DL, Coetzee SG, Henderson BE, Noushmehr H, Cozen W, Kote-Jarai Z (2014). Comprehensive functional annotation of 77 prostate cancer risk loci. PLoS genetics.

[9] Corradin O, Scacheri PC (2014). Enhancer variants: evaluating functions in common disease. Genome Med.

[10] Loven J, Hoke HA, Lin CY, Lau A, Orlando DA, Vakoc CR, Bradner JE, Lee TI, Young RA (2013). Selective inhibition of tumor oncogenes by disruption of super-enhancers. Cell.

[11] Whyte WA, Orlando DA, Hnisz D, Abraham BJ, Lin CY, Kagey MH, Rahl PB, Lee TI, Young RA (2013). Master transcription factors and mediator establish super-enhancers at key cell identity genes. Cell.

[12] Hnisz D, Abraham BJ, Lee TI, Lau A, Saint-Andre V, Sigova AA, Hoke HA, Young RA (2013). Super-enhancers in the control of cell identity and disease. Cell.

[13] Arshad Z, Smith J, Roberts M, Lee WH, Davies B, Bure K, Hollander GA, Dopson S, Bountra C, Brindley D (2016). Open Access Could Transform Drug Discovery: A Case Study of JQ1. Expert opinion on drug discovery.

[14] Asangani IA, Dommeti VL, Wang X, Malik R, Cieslik M, Yang R, Escara-Wilke J, Wilder-Romans K, Dhanireddy S, Engelke C (2014). Therapeutic targeting of BET bromodomain proteins in castration-resistant prostate cancer. Nature.

[15] Shaikhibrahim Z, Offermann A, Braun M, Menon R, Syring I, Nowak M, Halbach R, Vogel W, Ruiz C, Zellweger T (2014). MED12 overexpression is a frequent event in castration-resistant prostate cancer. Endocr Relat Cancer.

[16] Liu G, Sprenger C, Wu PJ, Sun S, Uo T, Haugk K, Epilepsia KS, Plymate S (2015). MED1 mediates androgen receptor splice variant induced gene expression in the absence of ligand. Oncotarget.

[17] Andreassen OA, Zuber V, Thompson WK, Schork AJ, Bettella F, Djurovic S, Desikan RS, Mills IG, Dale AM (2014). Shared common variants in prostate cancer and blood lipids. International journal of epidemiology.

[18] Schork AJ, Thompson WK, Pham P, Torkamani A, Roddey JC, Sullivan PF, Kelsoe JR, O'Donovan MC, Furberg H, Schork NJ (2013). All SNPs Are Not Created Equal: Genome-Wide Association Studies Reveal a Consistent Pattern of Enrichment among Functionally Annotated SNPs. PLoS genetics.

[19] Whitington T, Gao P, Song W, Ross-Adams H, Lamb AD, Yang Y, Svezia I, Klevebring D, Mills IG, Karlsson R (2016). Gene regulatory mechanisms underpinning prostate cancer susceptibility. Nature genetics.

[20] Psychiatric GWAS Consortium Bipolar Disorder Working Group. Large-scale genome-wide association analysis of bipolar disorder identifies a new susceptibility locus near ODZ4. Nature genetics 2011, 43(10):977–983. 10.1038/ng.943 PMC3637176 21926972

[21] Hoefer J, Kern J, Ofer P, Eder IE, Schäfer G, Dietrich D, Kristiansen G, Geley S, Rainer J, Gunsilius E (2014). SOCS2 correlates with malignancy and exerts growth-promoting effects in prostate cancer. Endocr Relat Cancer.

[22] Massie CE, Lynch A, Ramos-Montoya A, Boren J, Stark R, Fazli L, Warren A, Scott H, Madhu B, Sharma N (2011). The androgen receptor fuels prostate cancer by regulating central metabolism and biosynthesis. The EMBO journal.

[23] Wang D, Garcia-Bassets I, Benner C, Li W, Su X, Zhou Y, Qiu J, Liu W, Kaikkonen MU, Ohgi KA (2011). Reprogramming transcription by distinct classes of enhancers functionally defined by eRNA. Nature.

[24] Khan A, Zhang X (2016). dbSUPER: a database of super-enhancers in mouse and human genome. Nucleic acids research.

[25] Nagarajan S, Hossan T, Alawi M, Najafova Z, Indenbirken D, Bedi U, Taipaleenmaki H, Ben-Batalla I, Scheller M, Loges S (2014). Bromodomain protein BRD4 is required for estrogen receptor-dependent enhancer activation and gene transcription. Cell Rep.

[26] Theodorou V, Stark R, Menon S, Carroll JS (2013). GATA3 acts upstream of FOXA1 in mediating ESR1 binding by shaping enhancer accessibility. Genome research.

[27] Liu T, Ortiz JA, Taing L, Meyer CA, Lee B, Zhang Y, Shin H, Wong SS, Ma J, Lei Y (2011). Cistrome: an integrative platform for transcriptional regulation studies. Genome biology.

[28] He HH, Meyer CA, Chen MW, Jordan VC, Brown M, Liu XS (2012). Differential DNase I hypersensitivity reveals factor-dependent chromatin dynamics. Genome research.

[29] Eeles RA, Olama AA, Benlloch S, Saunders EJ, Leongamornlert DA, Tymrakiewicz M, Ghoussaini M, Luccarini C, Dennis J, Jugurnauth-Little S (2013). Identification of 23 new prostate cancer susceptibility loci using the iCOGS custom genotyping array. Nature genetics.

[30] Eeles RA, Kote-Jarai Z, Giles GG, Olama AA, Guy M, Jugurnauth SK, Mulholland S, Leongamornlert DA, Edwards SM, Morrison J (2008). Multiple newly identified loci associated with prostate cancer susceptibility. Nature genetics.

[31] Michailidou K, Hall P, Gonzalez-Neira A, Ghoussaini M, Dennis J, Milne RL, Schmidt MK, Chang-Claude J, Bojesen SE, Bolla MK (2013). Large-scale genotyping identifies 41 new loci associated with breast cancer risk. Nature genetics.

[32] Timofeeva MN, Hung RJ, Rafnar T, Christiani DC, Field JK, Bickeboller H, Risch A, McKay JD, Wang Y, Dai J (2012). Influence of common genetic variation on lung cancer risk: meta-analysis of 14 900 cases and 29 485 controls. Human molecular genetics.

[33] Lambert JC, Ibrahim-Verbaas CA, Harold D, Naj AC, Sims R, Bellenguez C, DeStafano AL, Bis JC, Beecham GW, Grenier-Boley B (2013). Meta-analysis of 74,046 individuals identifies 11 new susceptibility loci for Alzheimer's disease. Nature genetics.

[34] Taatjes DJ (2010). The human Mediator complex: a versatile, genome-wide regulator of transcription. Trends Biochem Sci.

[35] Heinz S, Romanoski CE, Benner C, Glass CK (2015). The selection and function of cell type-specific enhancers. Nature reviews Molecular cell biology.

[36] De Raedt T, Beert E, Pasmant E, Luscan A, Brems H, Ortonne N, Helin K, Hornick JL, Mautner V, Kehrer-Sawatzki H (2014). PRC2 loss amplifies Ras-driven transcription and confers sensitivity to BRD4-based therapies. Nature.

[37] Weickert CS, Weickert TW (2016). What's Hot in Schizophrenia Research?. The Psychiatric clinics of North America.

[38] Gusev A, Shi H, Kichaev G, Pomerantz M, Li F, Long HW, Ingles SA, Kittles RA, Strom SS, Rybicki BA (2016). Atlas of prostate cancer heritability in European and African-American men pinpoints tissue-specific regulation. Nature communications.

[39] Freedman ML, Monteiro AN, Gayther SA, Coetzee GA, Risch A, Plass C, Casey G, De Biasi M, Carlson C, Duggan D (2011). Principles for the post-GWAS functional characterization of cancer risk loci. Nature genetics.

[40] Polak P, Karlic R, Koren A, Thurman R, Sandstrom R, Lawrence MS, Reynolds A, Rynes E, Vlahovicek K, Stamatoyannopoulos JA (2015). Cell-of-origin chromatin organization shapes the mutational landscape of cancer. Nature.

