# Supplementary material for: Bromodomain protein 4 discriminates tissue-specific super-enhancers containing disease-specific susceptibility loci in prostate and breast cancer
Source: BMC Genomics. 2017 Mar 31;18:270. doi: 10.1186/s12864-017-3620-y (PMC5374680; doi:10.1186/s12864-017-3620-y)
Supplement: Supplementary file 22 — Including Supplementary Material such as Supplementary Figures S1–S11, Supplementary Tables S1–S5, and Supplementary References. (DOCX 2037 kb) [file 12864_2017_3620_MOESM22_ESM.docx]

**Additional file 22**

**Bromodomain protein 4 discriminates tissue-specific super-enhancers containing disease-specific susceptibility loci in prostate and breast cancer**

Verena Zuber^1,2,3,4^, Francesco Bettella^2,3^, Aree Witoelar^2,3^, the PRACTICAL Consortium^5§^, and the CRUK GWAS^§^, the BCAC Consortium^§^, the TRICL Consortium^§^, Ole A. Andreassen^2,3^, Ian G. Mills^1,6,7+^, Alfonso Urbanucci^1,6*+^

1 Prostate Cancer Research Group, Centre for Molecular Medicine Norway (NCMM), Nordic EMBL Partnership University of Oslo and Oslo University Hospital, Oslo, Norway;

2 NORMENT, KG Jebsen Centre for Psychosis Research, Institute of Clinical Medicine, University of Oslo, Oslo, Norway;

3 Division of Mental Health and Addiction, Oslo University Hospital, Oslo, Norway;

4 European Molecular Biology Laboratory, European Bioinformatics Institute, Wellcome Trust Genome Campus, Hinxton, Cambridge, UK;

5 Centre for Cancer Genetic Epidemiology, University of Cambridge, Cambridge, UK;

6 Department of Molecular Oncology, Institute of Cancer Research and Oslo University Hospital, Oslo, Norway;

7 PCUK Movember Centre of Excellence, CCRCB, Queen’s University, Belfast, UK;

§ The participants are fully acknowledged within this Supplementary Material

* Corresponding authors

+ Joint senior authors

**Content:**

**Supplementary Figures 1-11**

**Supplementary Tables 1-5**

**Supplementary References**

**Figure S1**

**
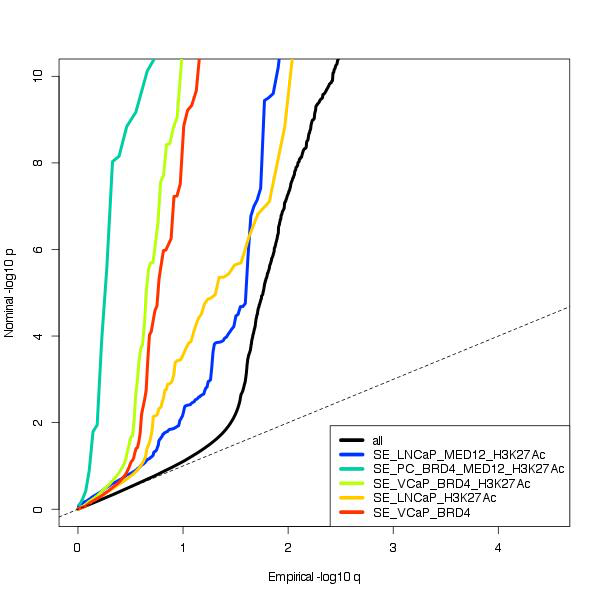
**

**Figure S1. Pruned Q-Q plots for prostate cancer associated SNPs *p*-values stratified according to affiliation to enhancers found in prostate cancer cells.** Q-Q plots visualizing the *p*-value enrichment of sets of pruned SNPs mapping within genomic intervals identified as regions of putative enhancers. The *p*-values describe the association of SNPs with prostate cancer (summary data provided by the iCOGs consortium). The genomic intervals represent regions bound by MED12, BRD4 with a H3K27Ac modification in prostate cancer cell lines as indicated in the legend.

**Figure S2**


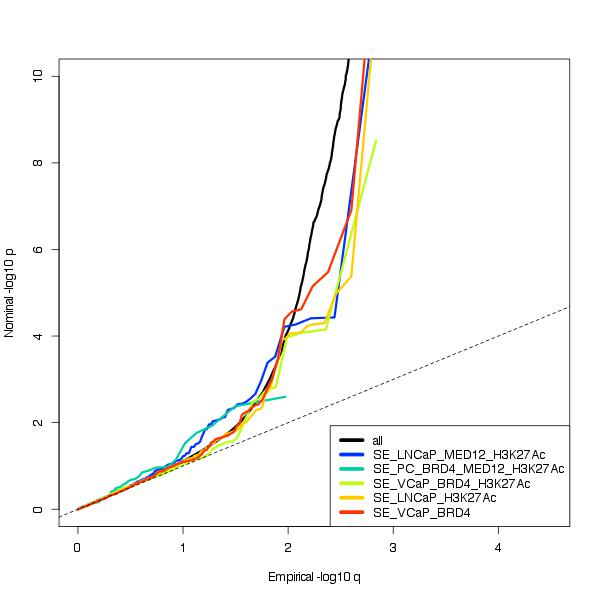


**Figure S2.** **Control enrichment of numerically matched SNPs.** Q-Q plots visualizing the *p*-value enrichment of SNP sets matching those found in the putative enhancers (indicated in the legend) on minor allele frequency and mutual LD r^2^.

**Figure S3**

**
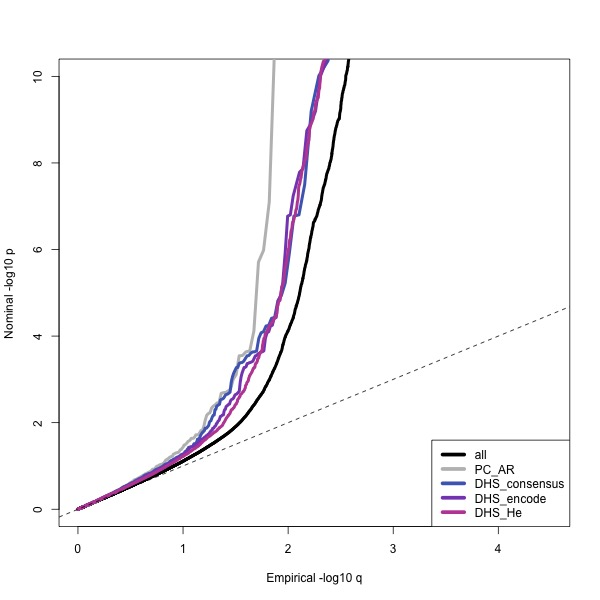
**

**Figure S3. Enrichment of SNPs lying within androgen receptor binding sites and DNase I hypersensitive sites.** Q-Q plots visualizing the *p*-value enrichment of sets of SNPs mapping within genomic intervals identified as regions of putative androgen receptor binding sites in LNCaP and VCaP cells (PC_AR) or DNase I hypersensitive sites (DHS) in LNCaP cells according to ENCODE (encode n= 20,405 SNPs), identified in He et al. (2012) [1] (n=164,260 SNPs), or in a intersect of those two (consensus n= 12,291 SNPs). The *p*-values describe the association of a specific SNP with prostate cancer according to summary statistics from iCOGs.

**Figure S4**


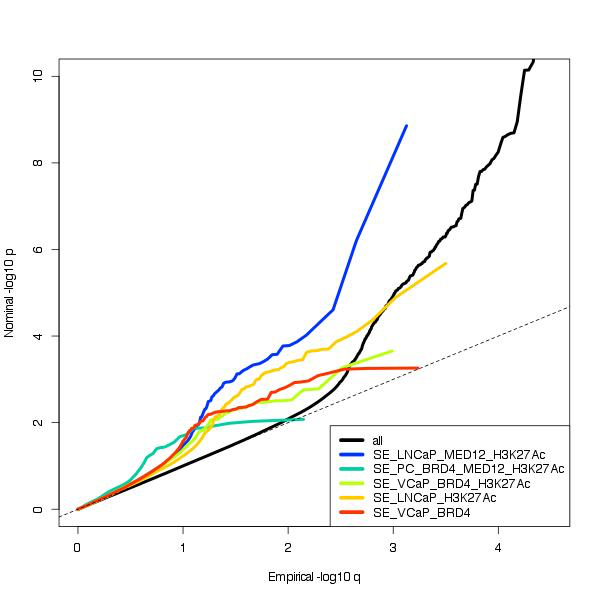


**Figure S4. Pruned Q-Q plots for association with breast cancer stratified according to enhancers found in prostate cancer cells.** Q-Q plots visualizing the *p*-value enrichment of sets of pruned SNPs mapping within genomic intervals identified as regions of putative enhancers or key transcription factor binding sites. The *p*-values describe the association of specific SNPs with breast cancer (summary data provided by the BCAC). The genomic intervals represent regions bound by MED12, BRD4 with a H3K27Ac modification in prostate cancer cell lines as indicated in the legend.

**Figure S5**


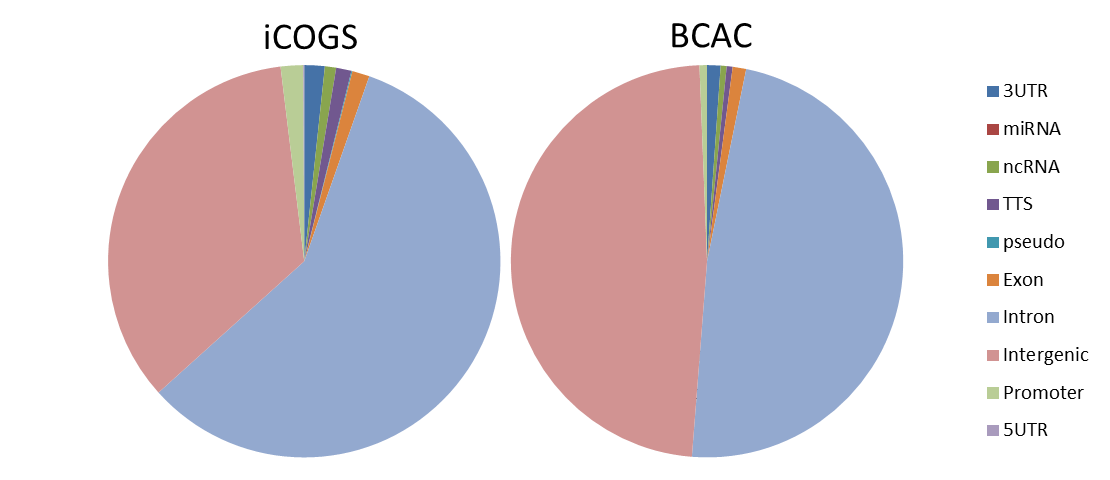


**Figure S5. Genomic distribution of inputed SNPs in iCOGS and BCAC array.** Distribution in annotated regions of the human genome for 1189 and 812 SNPs included in iCOGS and BCAC arrays, respectively.

**Figure S6**


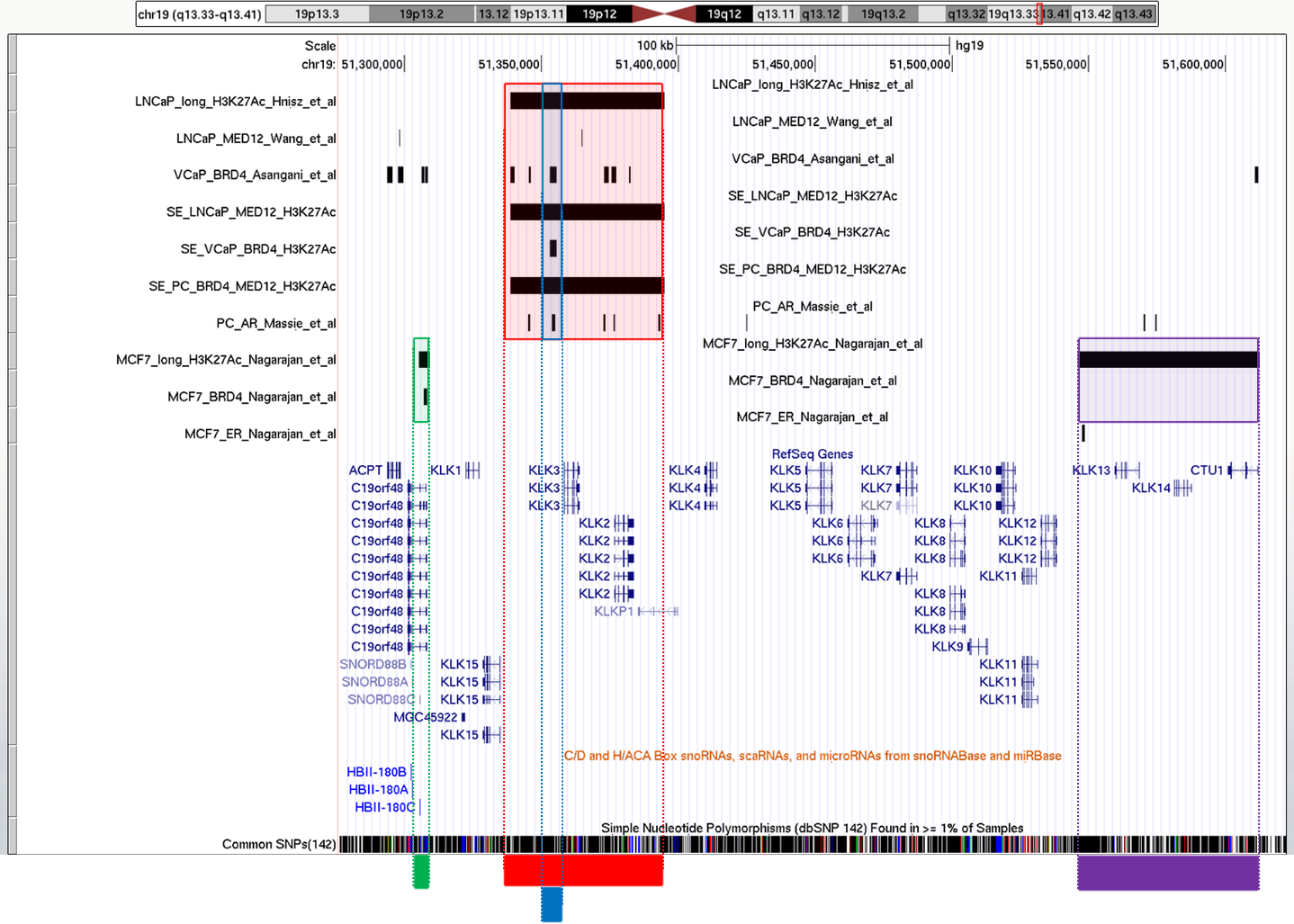


**Figure S6**. **Enhancers in prostate and breast cancer.** UCSC genome browser snapshot of the kallikreins locus showing enhancers identified in LNCaP based on MED12 binding information retrieved from Wang et al., (2012), and H3K27Ac profile retrieved from Hnisz et al., (2014)[2]; enhancers identified in VCaP based on BRD4 binding and H3K27Ac retrieved from Asangani et al., (2014)[3]; and common enhancers in prostate cancer (PC) identified selecting enhancers in LNCaP which also had BRD4 and acetylation signature according to the compendium of SEs in VCaP cells. Enhancers identified in MCF7 representing a breast cancer model are shown as an example here. Data on H3K27Ac profile, BRD4 and ER binding were retrieved from Nagarajan et al., (2014) [4] and are shown here to identify enhancers marked by H3K27Ac (purple) and enhancers marked by both BRD4 and H3K27Ac (green).

An independent track for ER binding sites in MCF7 cells according to Nagarajan et al., (2014) [4] is also shown.

**Figure S7**

**
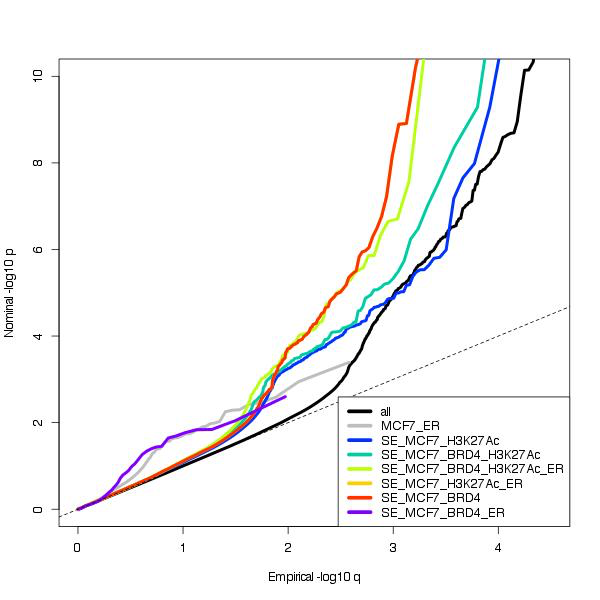
**

**Figure S7. Pruned Q-Q plots for association with breast cancer stratified according to affiliation to enhancers found in breast cancer cells.** Q-Q plots visualizing the *p*-value enrichment of sets of pruned SNPs mapping within genomic intervals identified as regions of putative enhancers or key transcription factor binding sites. The *p*-values describe the association of SNPs with breast cancer as measured in the BCAC consortium. The genomic intervals represent regions bound by BRD4 and/or ER with or without the H3K27Ac modification in MCF7 cells as indicated in the legend.

**Figure S8**


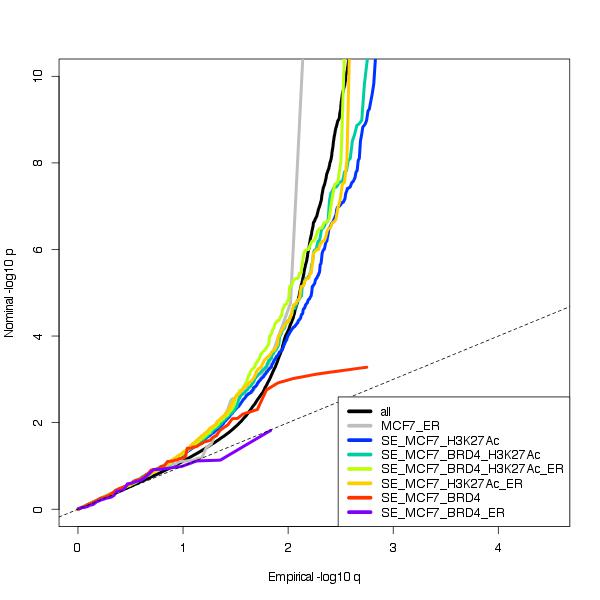


**Figure S8. Enrichment of prostate cancer associated SNPs lying within enhancers identified in breast cancer cells.** Q-Q plots visualizing the *p*-value enrichment of sets of SNPs mapping within genomic intervals identified as regions of putative enhancers or estrogen receptor (ER) binding sites in MCF7. *P-*values describe the association of a SNP with prostate cancer risk as measured in the iCOGs consortium. The genomic intervals represent regions bound by BRD4 and/or ER with or without H3K27Ac modification as indicated in the legends.

**Figure S9**


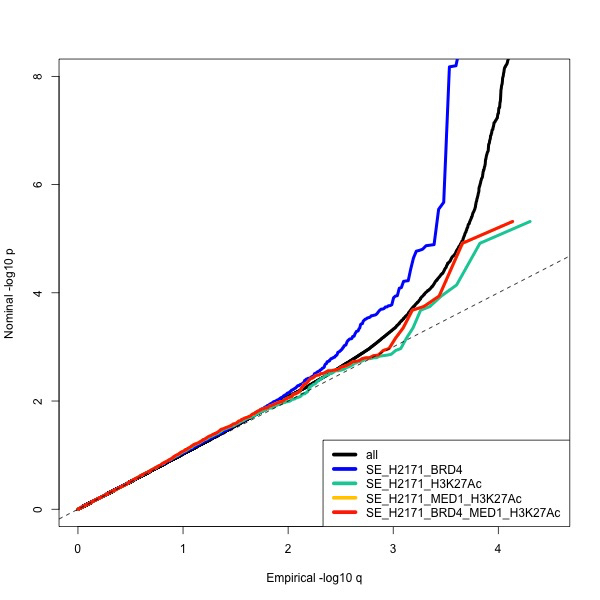


**a**

**b**


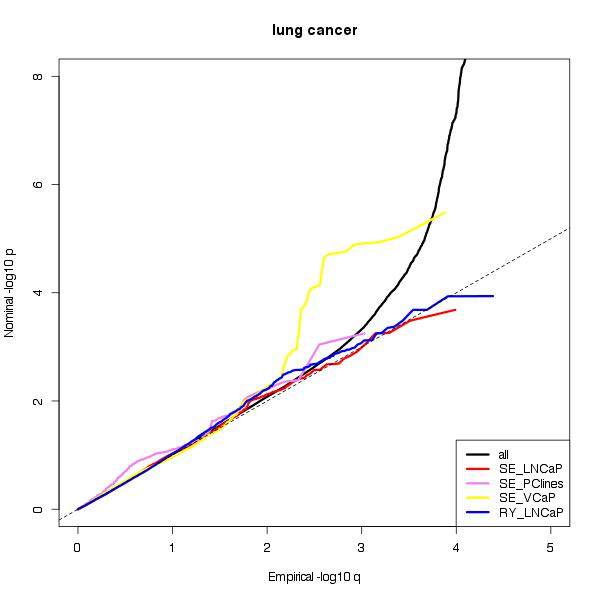

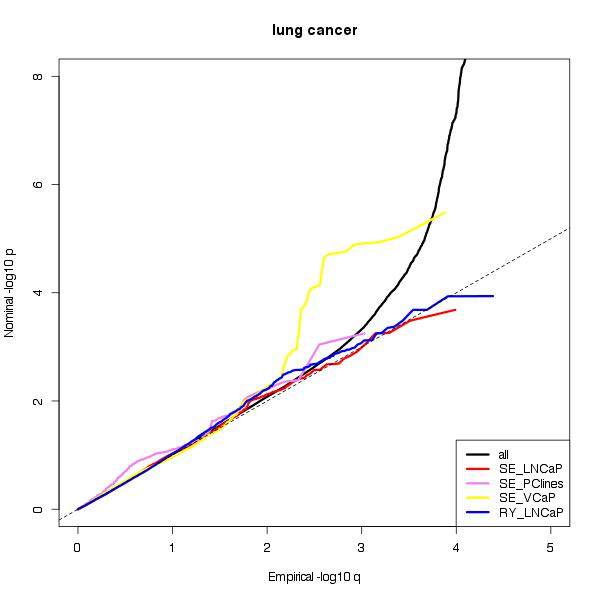

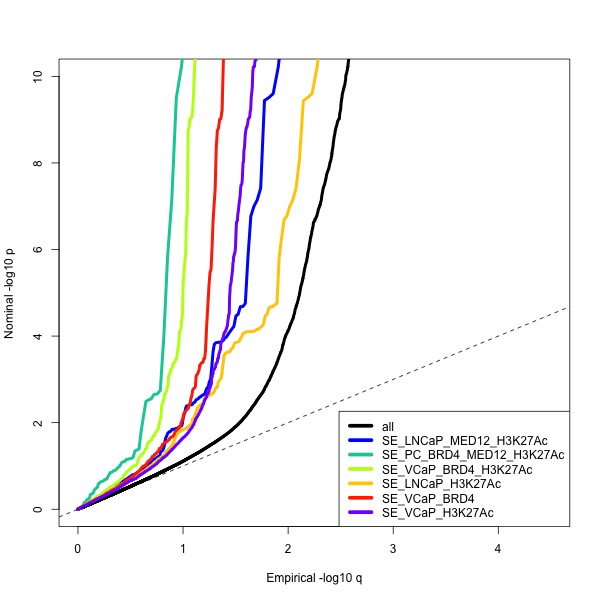


**Figure S9. Enrichment of lung cancer associated SNPs lying within enhancers identified in H2171 cells.** Q-Q plots visualizing the *p*-value enrichment of sets of SNPs mapping within genomic intervals identified as regions of putative enhancers in H2171 cells (**a**) or in prostate cancer cells LNCaP and VCaP (**b**). *P-*values describe the association of a specific SNP with lung cancer. The genomic intervals represent regions bound by BRD4 and/or MED1/MED12 with or without H3K27Ac modification as indicated in the legends. (RY_LNCaP= super-enhancers identified in LNCaP cells by the group of Richard A. Young).

**Figure S10**

**
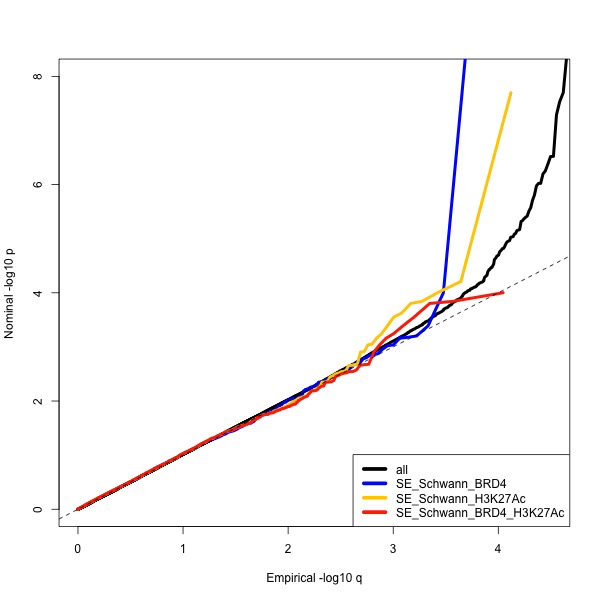
**

**a**

**b**

**
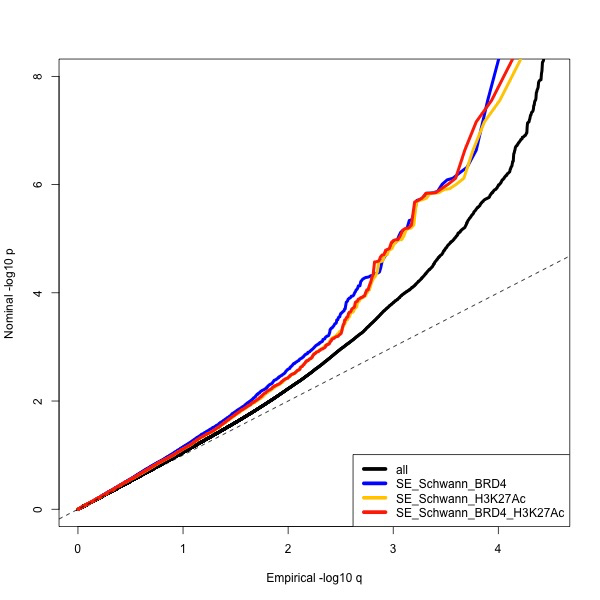
**

**Figure S10. Enrichment of brain-related diseases associated SNPs lying within enhancers identified in Schwann cells.** Q-Q plots visualizing the *p*-value enrichment of sets of SNPs mapping within genomic intervals identified as regions of putative enhancers in Schwann cells. *P-*values describe the association of a specific SNP with Alzheimer Disease (**a**) and Bipolar Disorder (**b**). The genomic intervals represent regions bound by BRD4 with or without H3K27Ac modification as indicated in the legends.

**Figure S11**


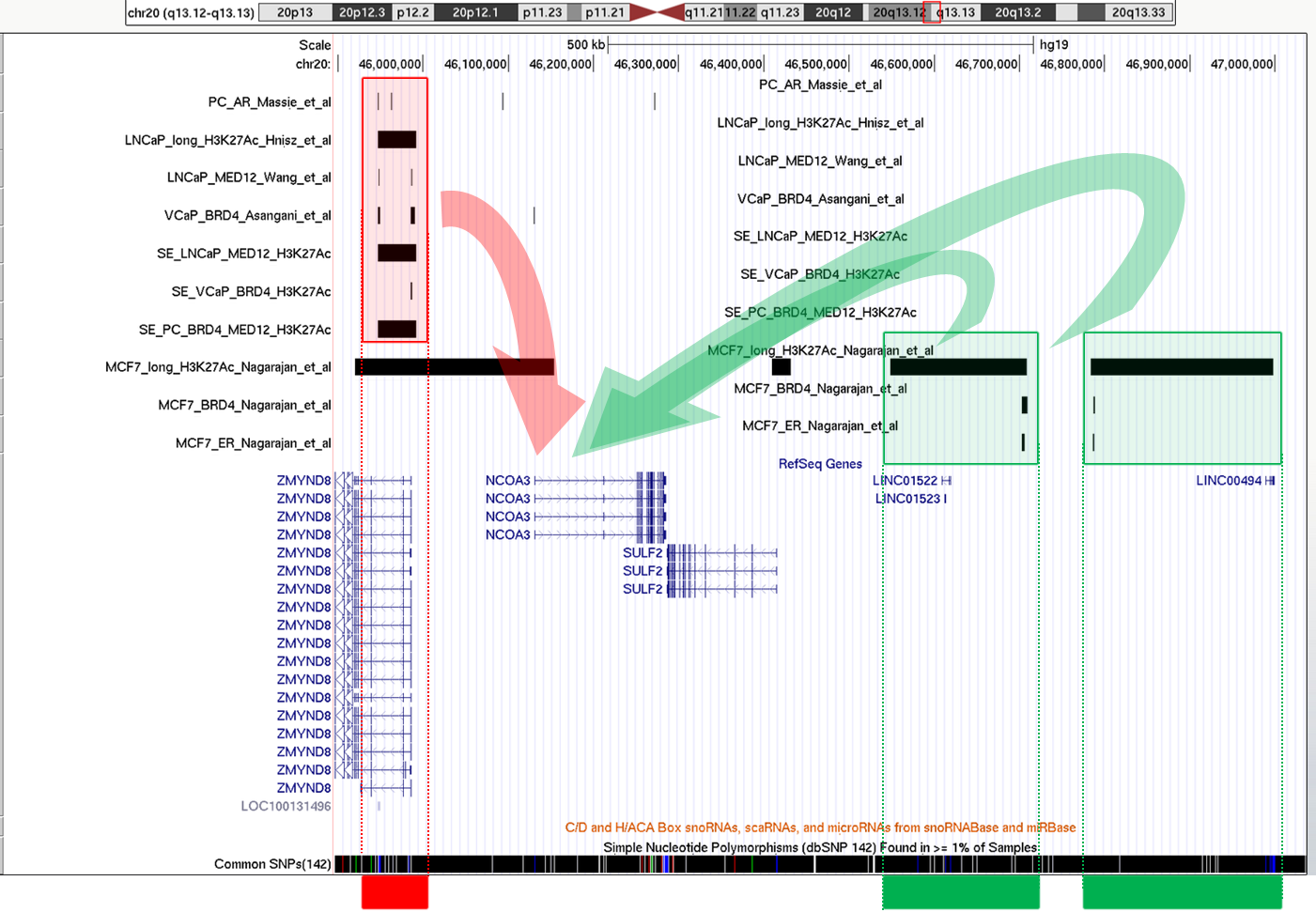


**Figure S11. Model of tissue specific super-enhancer usage.** UCSC genome browser snapshot showing the NCOA3 (AIB1) locus as a model. The NCOA3 gene is known to be AR regulated [5] and highly expressed in prostate cancer (PC) patients [6,7]. In breast cancer (BC) AIB1 expression correlates with estrogen receptor (ER) expression [6,8]. Based on our study, SNPs present in the vicinity of the gene locus that are not GWAS significant, may underlie susceptibility to develop breast or prostate cancer, based on the fact that this gene could be controlled by tissue specific super-enhancers (indicated in red for PC and in green for BC). In this view, BRD4 binding may represent the primary route of identification of such tissue-specific super-enhancers and risk SNPs contained within these loci.

**Table S1. Numbers of SNPS that are GWAS significant (-log10 *p*-value <7.3) in the sets of SNPs identified in Figure 1a**

|  | LNCaP_MED12_H3K27Ac | PC_BRD4_MED12_H3K27Ac | VCaP_BRD4_H3K27Ac | LNCaP_H3K27Ac | SE_VCaP_BRD4 |
| --- | --- | --- | --- | --- | --- |
| iCOGS | 13/685* | 10/82* | 53/587* | 14/1605 | 43/859* |
| PRACTICAL | 4/1271* | 4/113* | 1/938 | 4/392* | 1/1595 |
| BCAC | 9/5428* | 0/618 | 0/4218 | 9/13482* | 0/7984 |

*** significant at a level of 0.05 (after correction for multiple testing) according to Fisher’s hypergeometric test.**

**Table S2. GWAS significant SNPs in iCOGs captured by super-enhancers featuring H3K27Ac, MED12, and BRD4 binding in prostate cancer cells**

| **SNP rs number** | **- Log-transformed p-value according to iCOGs** |
| --- | --- |
| rs2659051 | 13,701 |
| rs266849 | 11,413 |
| rs266863 | 9,5047 |
| rs266878 | 14,746 |
| rs174776 | 15,358 |
| rs17632542 | 22,204 |
| rs2659122 | 10,217 |
| rs1058205 | 12,17 |
| rs2569735 | 13,788 |
| rs2735839 | 13,91 |

**Table S3. Numbers of SNPS that are GWAS significant (-log10 *p*-value <7.3) in the sets of SNPs identified in Figure 1b.**

|  | PC_AR | LNCaP_MED12_H3K27Ac_AR | PC_BRD4_MED12_H3K27Ac_AR | VCaP_BRD4_H3K27Ac_AR | LNCaP_H3K27Ac_AR | VCaP_BRD4_AR |
| --- | --- | --- | --- | --- | --- | --- |
| iCOGS | 7/496 | 14/669* | 10/49* | 53/342* | 13/279* | 1/23 |
| PRACTICAL | 1/1403 | 4/1274 | 4/46 | 1/502 | 4/541 | 0/60 |
| BCAC | 2/5950 | 0/5664 | 0/248 | 0/2148 | 0/2310 | 0/233 |

*** significant at a level of 0.05 (after correction for multiple testing) according to Fisher’s hypergeometric test.**

**Table S4. Numbers of SNPS that are GWAS significant (-log10 *p*-value <7.3) in the sets of SNPs identified in Figure 1e.**

|  | MCF_ER | MCF7_H3K27Ac | MCF7_BRD4_H3K27Ac | MCF7_BRD4_H3K27Ac_ER | MCF7_H3K27Ac_ER | MCF7_BRD4 | MCF7_BRD4_ER |
| --- | --- | --- | --- | --- | --- | --- | --- |
| iCOGS | 1/158 | 54/19270 | 34/8783 | 16/4296 | 21/6710 | 0/280 | 0/34 |
| PRACTICAL | 0/371 | 1/48028 | 0/21058 | 0/10607 | 1/16206 | 0/495 | 0/66 |
| BCAC | 1/1638 | 88/215997* | 53/93969* | 42/45937* | 58/69743* | 12/2617* | 0/282 |

*** significant at a level of 0.05 (after correction for multiple testing) according to Fisher’s hypergeometric test.**

**Table S5. GWAS significant SNPs in BCAC captured by BRD4 binding in breast cancer cells**

| **SNP rs number** | **- Log-transformed p-value according to BCAC** |
| --- | --- |
| rs9397435 | 12,85 |
| rs9397436 | 13,042 |
| rs9397437 | 13,148 |
| rs6900157 | 11,331 |
| rs9383937 | 13,162 |
| rs10484919 | 10,146 |
| rs6915267 | 10,147 |
| rs9371547 | 10,28 |
| rs9479090 | 11,087 |
| rs614367 | 16,934 |
| rs661204 | 21,189 |
| rs554219 | 21,501 |

**Supplementary References**

1. He HH, Meyer CA, Chen MW, Jordan VC, Brown M, et al. (2012) Differential DNase I hypersensitivity reveals factor-dependent chromatin dynamics. Genome Res 22: 1015-1025.

2. Hnisz D, Abraham BJ, Lee TI, Lau A, Saint-Andre V, et al. (2013) Super-enhancers in the control of cell identity and disease. Cell 155: 934-947.

3. Asangani IA, Dommeti VL, Wang X, Malik R, Cieslik M, et al. (2014) Therapeutic targeting of BET bromodomain proteins in castration-resistant prostate cancer. Nature 510: 278-282.

4. Nagarajan S, Hossan T, Alawi M, Najafova Z, Indenbirken D, et al. (2014) Bromodomain protein BRD4 is required for estrogen receptor-dependent enhancer activation and gene transcription. Cell Rep 8: 460-469.

5. Urbanucci A, Waltering KK, Suikki HE, Helenius MA, Visakorpi T (2008) Androgen regulation of the androgen receptor coregulators. BMC Cancer 8: 219.

6. Gojis O, Rudraraju B, Alifrangis C, Krell J, Libalova P, et al. (2010) The role of steroid receptor coactivator-3 (SRC-3) in human malignant disease. Eur J Surg Oncol 36: 224-229.

7. Zhou HJ, Yan J, Luo W, Ayala G, Lin SH, et al. (2005) SRC-3 is required for prostate cancer cell proliferation and survival. Cancer Res 65: 7976-7983.

8. Chang AK, Wu H (2012) The role of AIB1 in breast cancer. Oncol Lett 4: 588-594.
